# Mesenchymal stem cells alleviate LPS-induced acute lung injury by inhibiting the proinflammatory function of Ly6C^+^ CD8^+^ T cells

**DOI:** 10.1038/s41419-020-03036-1

**Published:** 2020-10-06

**Authors:** Jiaqi Zhu, Bing Feng, Yanping Xu, Wenyi Chen, Xinyu Sheng, Xudong Feng, Xiaowei Shi, Jingqi Liu, Qiaoling Pan, Jiong Yu, Lanjuan Li, Hongcui Cao

**Affiliations:** 1 State Key Laboratory for the Diagnosis and Treatment of Infectious Diseases, The First Affiliated Hospital, College of Medicine, Zhejiang University, 79 Qingchun Road, Hangzhou City, 310003 China; 2 National Clinical Research Center for Infectious Diseases, 79 Qingchun Road, Hangzhou City, 310003 China; 3 Zhejiang Provincial Key Laboratory for Diagnosis and Treatment of Aging and Physic-chemical Injury Diseases, 79 Qingchun Road, Hangzhou City, 310003 China

**Keywords:** Mesenchymal stem cells, Experimental models of disease

## Abstract

Systemic inflammatory processes, including alveolar injury, cytokine induction, and neutrophil accumulation, play key roles in the pathophysiology of acute lung injury (ALI). The immunomodulatory effects of mesenchymal stem cells (MSCs) can contribute to the treatment of inflammatory disorders. In previous studies, the focus was on innate immune cells and the effects of MSCs on ALI through CD8^+^ T cells remain unclear. In the present study, lipopolysaccharide (LPS) was used to induce ALI in mice. ALI mice were treated with MSCs via intratracheal instillation. Survival rate, histopathological changes, protein levels, total cell count, cytokine levels, and chemokine levels in alveolar lavage fluid were used to determine the efficacy of MSCs. Mass cytometry and single-cell RNA sequencing (scRNA-seq) were used to characterize the CD8^+^ T cells in the lungs. Ly6C^−^ CD8^+^ T cells are prevalent in normal mice, whereas a specialized effector phenotype expressing a high level of Ly6C is predominant in advanced disease. MSCs significantly mitigated ALI and improved survival. MSCs decreased the infiltration of CD8^+^ T cells, especially Ly6C^+^ CD8^+^ T cells into the lungs. Mass cytometry revealed that CD8^+^ T cells expressing high Ly6C and CXCR3 levels caused tissue damage in the lungs of ALI mice, which was alleviated by MSCs. The scRNA-seq showed that Ly6C^+^ CD8^+^ T cells exhibited a more activated phenotype and decreased expression of proinflammatory factors that were enriched the most in immune chemotaxis after treatment with MSCs. We showed that CD8^+^ T cells play an important role in MSC-mediated ALI remission, and both infiltration quantity and proinflammatory function were inhibited by MSCs, indicating a potential mechanism for therapeutic intervention.

## Introduction

Acute lung injury (ALI) and acute respiratory distress syndrome (ARDS) are a continuum of lung changes caused by multiple lung injuries, often resulting in severe morbidity and death^1,2^. These diseases lead to respiratory failure, increase susceptibility to multiple organ dysfunction, and are a common cause of death in critically ill patients of all ages^3^. ALI is a severe pulmonary inflammatory disease characterized by diffuse interstitial and alveolar edema, inflammatory cell infiltration, and the release of proinflammatory factors^4–7^. Direct intratracheal infusion of lipopolysaccharide (LPS) is commonly used to study pulmonary inflammation and ALI in small animal models, such as mice^8^. Attenuation of alveolar inflammation and recovery of barrier function contribute to an improved prognosis^9^. Mesenchymal stem cells (MSCs) are multipotent stromal cells that have potential for cell therapy because of their advantages of pluripotency in vitro, low immunogenicity, and tumorigenicity^10^. MSCs have potential for use in the treatment of ALI due to their immunosuppressive effects. An increasing number of preclinical studies support the transplantation of MSCs for treatment of ALI^11,12^. Furthermore, MSCs can alleviate acute inflammation by interacting with the immune system^13^. However, the underlying mechanisms of treatment have not yet been thoroughly explored.

MSCs have significant immunoregulatory capacity, especially in the adaptive immune system, and could be used in the treatment of inflammatory diseases. MSCs have been reported to regulate many aspects of the T-cell response, including proliferation, survival, and differentiation^14^. In previous studies, MSCs were shown to reduce CD8^+^ cytotoxic T cells via stanniocalcin-2 and to alleviate the inflammatory reaction in mice^15^. Ronit et al.^16^ reported the recruitment of CD8^+^ T cells following LPS-induced lung injury. Risso et al.^17^ discovered that CD8^+^ T cells showed a significantly activated phenotype in ALI/ARDS compared with the control group. However, knowledge regarding the effects of MSC treatment on CD8^+^ T cells in ALI mice is limited. We found significant infiltration of Ly6C^+^ CD8^+^ T cells during ALI progression, which was suppressed by the addition of MSCs. Ly6C is a member of the Ly6 family, a type of surface molecule that is differentially expressed in a variety of immune cells^18^. Kusaka et al.^19^ discovered that Ly6C^+^ CD8^+^ T cells are a source of interferon-γ (IFN-γ) during the acute phase of infection. In the present study, whether MSC transplantation reduced the risk of death in ALI mice by affecting Ly6C^+^ CD8^+^ T cells was investigated.

## Materials and methods

### Mouse model of ALI treated with MSCs

Healthy, male, wild-type (WT) C57BL/6 mice (6–8 weeks of age) were provided by the Nanjing Biomedical Research Institute of Nanjing University, Nanjing, China. The mice were randomly distributed into three groups. The ALI model was induced by intratracheal inhalation of 20 mg/kg LPS (Sigma, Beijing, China). After 4 h, the LPS/MSC group received intratracheal inhalation of 5 × 10^5^ MSCs resuspended in 20 μL phosphate-buffered saline (PBS) (containing 2% mouse serum) and the LPS/PBS group received only 20 μL PBS (containing 2% mouse serum). The PBS/PBS group underwent the same treatment, except that LPS and MSCs were replaced by PBS. The sample size was determined based on the survival rate results. On days 3 and 7 after intratracheal inhalation, three to six mice per group per time point were anesthetized and sampled. The double-blind method was used in the experiment. All experiments were conducted using protocols approved by the Animal Care Ethics Committee of the First Affiliated Hospital, Zhejiang University.

### Extraction and detection of BALF

The experimental mice were anesthetized with 4% chloral hydrate (Sangon Biotech) and an intravenous trocar was inserted into the trachea to collect bronchoalveolar lavage fluid (BALF), followed by two flushes with 0.8 mL PBS. Pulmonary dilation was observed and > 80% PBS was recovered. The collected BALF was centrifuged at 300 *×* *g* for 5 min at 4 °C and the supernatant was dispensed into aliquots and kept at −80 °C for subsequent assay of cytokines, chemokines, and protein concentration. The diluted cells were distributed on cell-counting plates and counted under a microscope. For differential cell sorting, cells were stained with Wright-Giemsa reagents (Baso, Zhuhai, China). The number of neutrophils, macrophages, and lymphocytes per 200 cells was determined based on morphology. Cytokines and chemokines were measured using the LEGENDplex^TM^ Multi-Analyte Flow Assay Kit (Biolegend). BALF protein concentration was measured using the BCA Protein Assay Kit (Sangon Biotech).

### Lung tissue histology

Lung specimens were fixed in 4% paraformaldehyde, embedded in paraffin, sliced into 5 μm-thick sections, and stained with hematoxylin and eosin according to a standard methodology. Areas of particular concern were analyzed using a NanoZoomer 2.0-RS scanner (Hamamatsu, Shizuoka, Japan).

### Isolation of immune cells for mass cytometry and scRNA-seq

Mice were anesthetized with 4% chloral hydrate; heart perfusion was performed until the lungs turned pale, which were then removed and cut into pieces. The mouse Lung Dissociation Kit (Miltenyi Biotec, Bergisch Gladbach, Germany) was used for lung digestion. Filtration, density gradient centrifugation purification, and erythrocyte lysis were performed to obtain purified mouse lung immune cells. Single-cell suspensions were purified using mouse CD45 MicroBeads (Miltenyi Biotec) to collect CD45^+^ immune cells. Twenty-five mice were used for mass cytometry analysis and five for single-cell RNA sequencing (scRNA-seq).

### Mass cytometry marker labeling and data analysis

Metal isotope-tagged antibodies (Appendix Table S1) were used to evaluate the CD8^+^ cell populations in the mouse lungs. Antibody conjugation with the indicated metal tags, cell staining, and data acquisition were performed as previously described^20^. Briefly, antibody conjugation with the indicated metal tags was performed using the Maxpar X8 Antibody Conjugation Kit (Fluidigm Corp., San Francisco, CA, USA). The single lung cells were washed once in 1 mL fluorescence-activated cell sorting (FACS) buffer (PBS with 0.5% bovine serum albumin and 0.02% NaN_3_) and incubated with 0.25 μM cisplatin (Fluidigm Corp.) on ice for 5 min to discriminate the dead cells. The Fc receptors were blocked with 20 mg/mL mouse/hamster/rat total IgG (Equitech-Bio, Inc., Kerrville, TX, USA). The primary anti-CD49a-APC antibody (100 μL) was incubated with the cells on ice for 30 min; then, the cells were stained with a heavy metal isotope-labeled antibody cocktail (100 μL) on ice for 30 min. After incubation with 0.03 μM Ir nucleic-acid intercalator (Fluidigm Corp.) in Fix and Perm Buffer (Fluidigm Corp.) at 4 °C overnight, the cells were washed with Perm Buffer (eBioscience, Inc., San Diego, CA, USA) once and stained with a heavy metal isotope-labeled intracellular antibody cocktail (100 μL) in Perm buffer on ice for 30 min. The cells were resuspended at 0.6 × 10^6^/mL in distilled water containing 20% EQ. Four beads (Fluidigm Corp.) were filtered through capFACS tubes (Corning, Inc., Corning, NY, USA) and acquired using the Helios system (Fluidigm Corp.) under 500 events/s.

Raw mass cytometry data in.fcs files were pre-gated on live, single, valid CD45^+^ CD19^−^ CD3^+^ CD8^+^ cells and exported as.fcs files using FlowJo. In addition, FlowJo was used to visualize biaxial marker expression. The data were analyzed using R (version 3.6.1; R Development Core Team, Vienna, Austria); 1000 cells were randomly selected from each sample for downstream analysis. As the mass cytometry data were nonlinear, cytofAsinh was used for data normalization. After transformation, the data were analyzed using the PhenoGraph R package for clustering and the t-distributed stochastic neighbor embedding (t-SNE) algorithm was then applied to visualize the high-dimensional data in two dimensions. A heatmap of normalized marker expression levels was generated. The ggplo2 R package was used to display the data.

### scRNA-seq data processing and analysis

For scRNA-seq, cell suspensions with survival rates > 95% were diluted to 10^6^/mL and captured with the 10× Genomics platform (Pleasanton, CA, USA). The water-in-oil structure was used to capture single cells. In the rational case, a droplet could only hold one cell and one gel bead with barcode information. After cell lysis and 10× labeling of cDNA fragments, followed by amplification, library construction, and sequencing using an Illumina sequencer (San Diego, CA, USA), the raw sequencing data were obtained for subsequent analysis. The raw sequencing data were processed using the Cell Ranger pipeline (10× Genomics) and compared with the reference genome annotated using the Ensembl gene annotation system. The processed data were analyzed using the Seurat R package for single-cell genomics (https://satijalab.org/seurat/). Cell quality was assessed based on the following metrics: (1) total unique molecular identifier counts per cell; (2) number of detected genes per cell; and (3) ratio of mitochondrial genes. High-quality cells were reserved if there were 200–5000 detected genes and the proportion of mitochondrial genes was <15%. After dimensionality reduction by principal component analysis, clustering by graph-based clustering method, t-SNE, and uniform manifold approximation and projection (UMAP) were used for visualization. A total of 1638 cells that expressed CD8a in the cluster representing T cells were selected for further dimensional clustering analysis (Appendix Fig. S3). The Monocle R package for single-cell genomics was used for analysis of single-cell trajectory, which allowed cells to be arranged in simulated chronological order (http://cole-trapnell-lab.github.io/monocle-release/). MAST, a method for differential expression analysis of single-cell transcriptome sequencing data, was used^21^. Significantly differentially expressed genes (DEGs) were screened based on fold change > 1.5 and *P* < 0.05. Gene Ontology (GO) and Kyoto Encyclopedia of Genes and Genomes enrichment analyses were performed for the selected DEGs. The protein–protein interaction (PPI) network of DEGs was retrieved from the STRING database^22^ and reconstructed using Cytoscape software^23^. A plugin of Cytoscape, CytoHubba, was used to predict the top ten hub genes based on the maximal clique centrality (MCC) algorithm in the network^24^. The raw sequence data were submitted to the Genome Sequence Archive^25^ of the Beijing Institute of Genomics Data Center^26^, Chinese Academy of Sciences (accession number CRA003097), and are publicly available at https://bigd.big.ac.cn/gsa.

### Statistical analysis

Data are presented as means ± SD. The experiments presented in this research were replicated at least three times in the laboratory. The unpaired two-tailed *t*-test and Kaplan–Meier analysis were used for comparing two groups. Volcano plots of differentially abundant clusters among the groups were calculated using unpaired *t*-test analysis. The data analysis was performed using SPSS software (version 21.0; SPSS, Inc., Chicago, IL, USA). Differences were considered statistically significant when the *P*-value < 0.05, unless otherwise noted.

## Results

### MSCs ameliorated LPS-induced ALI

**Fig. 1:**
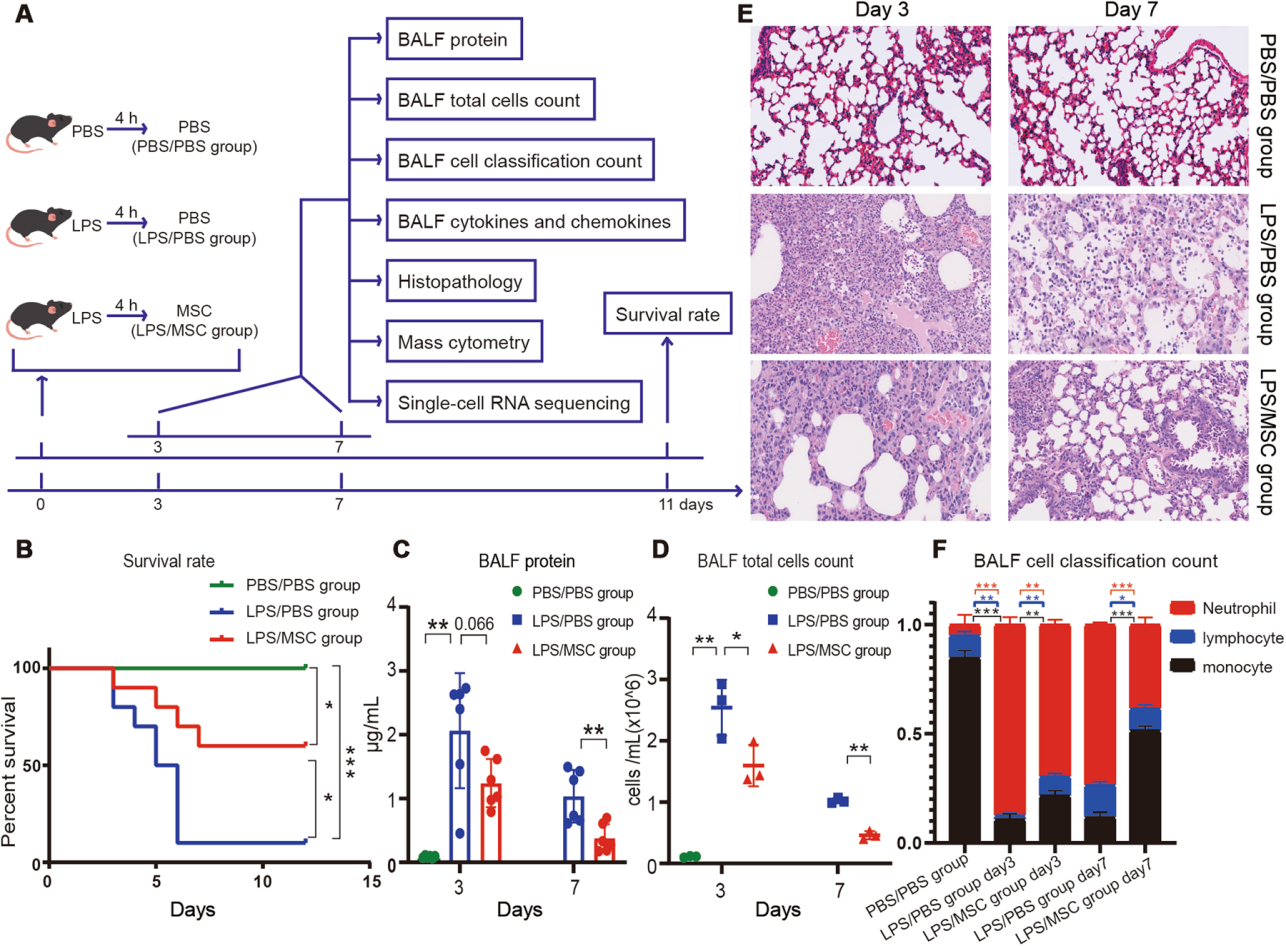
MSCs ameliorate LPS-induced ALI. **a** Schematic diagram of MSC treatment of ALI. **b** Survival curves of mice in the PBS/PBS, LPS/PBS, and LPS/MSC groups (*n* = 10, *P < 0.05 and ***P < 0.001 based on Kaplan–Meier analysis). **c** BALF total protein was determined in ALI mice after treatment with MSCs (*n* = 6, *P < 0.05 and **P < 0.01 based on unpaired Student’s *t*-test). **d** Total cell counts in BALF (*n* = 3, *P < 0.05 and **P < 0.01 based on unpaired Student’s *t*-test). **e** Hematoxylin and eosin (H&E)-stained images (×40 magnification) of the lungs. **f** The percentage changes of neutrophils, lymphocytes, and monocytes in BALF during ALI progression (*n* = 3, *P < 0.05, **P < 0.01, and ***P < 0.001 based on unpaired Student’s *t*-test).

Isolated MSCs were used to treat LPS-induced ALI. To determine how MSCs ameliorate LPS-induced ALI, mice were divided into three groups: PBS/PBS, LPS/PBS, and LPS/MSC (Fig. 1a). All mice were followed up for 11 days and the survival rates were recorded. The survival rate of mice in the LPS/MSC group was significantly higher than in the LPS/PBS group; no mice died in the PBS/PBS group (Fig. 1b).

The degree of lung injury was assessed based on the BALF protein concentration (Fig. 1c), BALF total cell count (Fig. 1d), lung histology (Fig. 1e), and BALF cell classification (Fig. 1f). The BALF protein level peaked at day 3 and then gradually decreased at day 7 after PBS or MSC treatment. The levels were markedly lower in the LPS/MSC group than in the LPS/PBS group, although the difference was not statistically significant at day 3 (Fig. 1c). BALF total cell count peaked in the LPS/PBS and LPS/MSC groups at day 3, decreased at day 7, and was lower in the LPS/MSC group than in the LPS/PBS group (Fig. 1d). After MSC or PBS administration, typical histopathological changes were observed under a microscope. Tissue from control mice showed no significant inflammation or cellular infiltration in the pulmonary alveoli. Sections from the LPS/PBS group showed neutrophilic alveolar and interstitial infiltration at day 3. In the LPS/MSC group, cellular infiltration was markedly ameliorated at day 3. On day 7, histological examination showed that the tissue had almost returned to normal in the LPS/MSC group; however, some interstitial infiltration and individual neutrophilic alveolar infiltration were observed in the lungs of the LPS/PBS group (Fig. 1e). The alveolar neutrophils peaked in both groups on day 3 (Fig. 1f), decreased by day 7 in the LPS/MSC group, but remained high in the LPS/PBS group.

In summary, these results indicated that lung injury was severe during ALI and significantly alleviated after administration of MSCs.

### Transplantation of MSCs into LPS-induced ALI mice reduced inflammatory factors

**Fig. 2:**
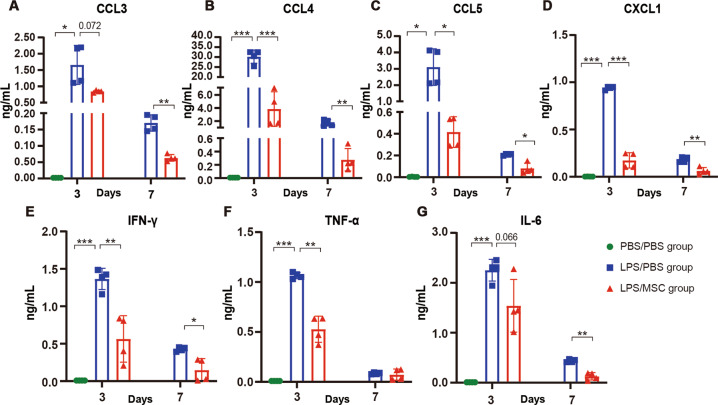
LEGENDplex^TM^ multi-analyte flow assay of inflammatory factors in BALF. LEGENDplex^TM^ multi-analyte flow assay of **a** CCL3, **b** CCL4, **c** CCL5, **d** CXCL1, **e** IFN-γ, **f** TNF-α and **g** IL6 in BALF from mice in the PBS/PBS, LPS/PBS, and LPS/MSC groups at days 3 and 7. Data are expressed as mean ± SD (*n* = 4, *P < 0.05, **P < 0.01, and ***P < 0.001 based on unpaired Student’s *t*-test).

The concentration of inflammatory factors was significantly increased in the BALF of mice treated with LPS, but was lower in the BALF of LPS/MSC mice compared with LPS/PBS mice, including CCL3, CCL4, CCL5, CXCL1, IFN-γ, TNF-α, and IL6 (Fig. 2). These results indicated that MSCs inhibited increased expression of cytokines and chemokines in the mouse lungs.

### MSCs downregulated Ly6C^+^ CD8^+^ T cells in ALI

**Fig. 3:**
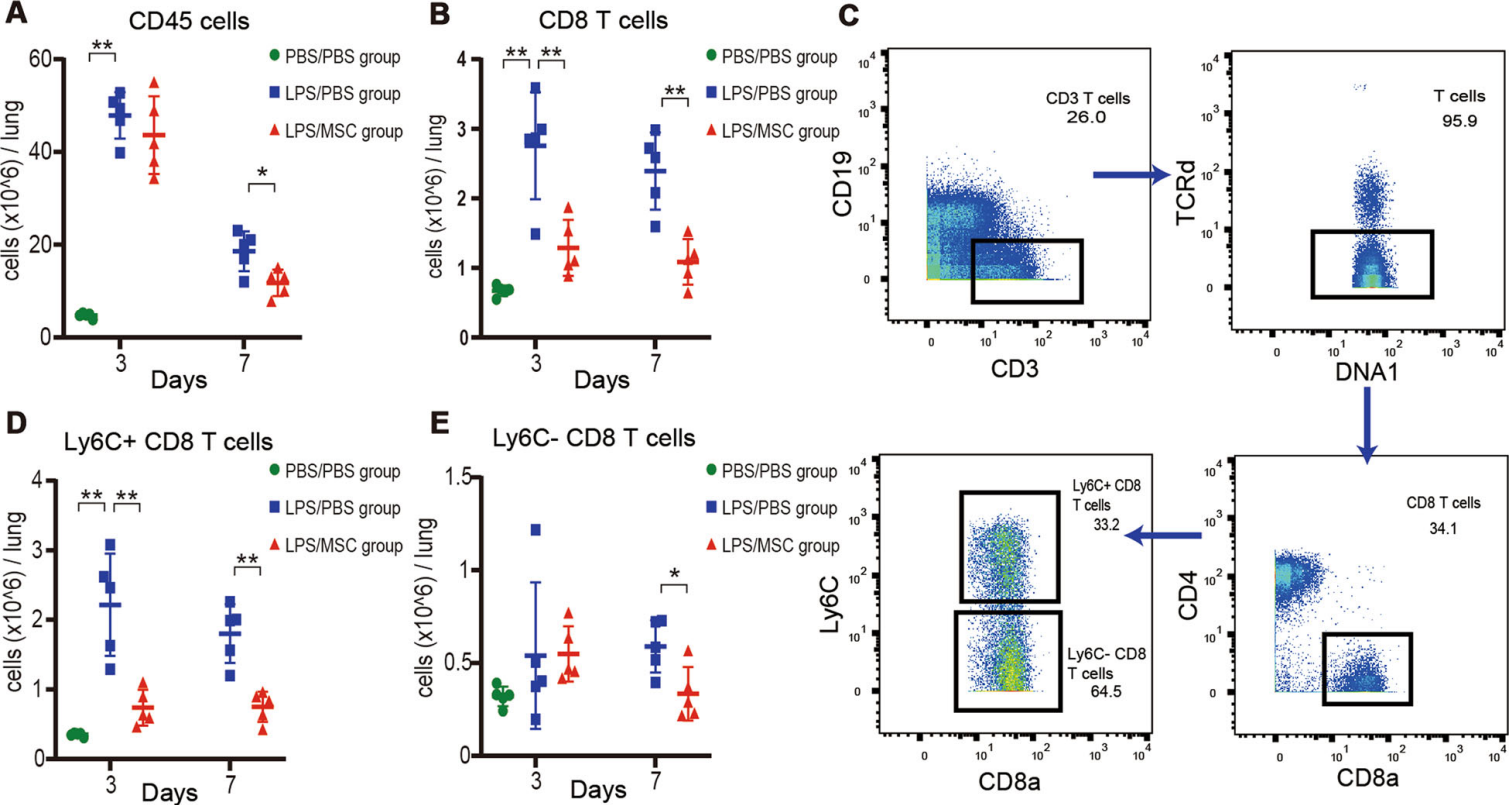
MSCs ameliorate Ly6C^+^ CD8^+^ T-cell infiltration. Absolute numbers of **a**. CD45^+^ immune cells (n = 5) and **b**. CD8^+^ T cells (n = 5) in mouse lung over time. **c**. Mass cytometry analysis of Ly6C^+^ and Ly6C^−^ CD8^+^ T-cell subsets defined based on manual gating strategy for a representative mouse. Absolute numbers of **d**. Ly6C^+^ CD8^+^ T cells (n = 5) and **e**. Ly6C^−^ CD8^+^ T cells (n = 5) in mouse lung over time. *P < 0.05, **P < 0.01, unpaired Student’s *t*-test.

CD45^+^ immune cells were isolated from the lungs. Cell count analysis demonstrated increased CD45^+^ infiltration as ALI developed, which decreased by day 7 with MSC treatment (Fig. 3a). Mass cytometry was used to analyze immune cells from the LPS-induced mice treated with MSCs or PBS. The mass cytometry data were analyzed and the CD8^+^ T cells gated using a rational strategy. A similar trend was found in the number of CD8^+^ cells with progression of ALI, with a lower number of cells in the LPS/MSC group at days 3 and 7 (Fig. 3b).

CD8^+^ T cells can be divided into two subsets: Ly6C^+^ and Ly6C^−^ (Fig. 3c). In WT mice, Ly6C^−^ CD8 T cells were the predominant cells. In mice with ALI, the proportion of Ly6C^+^ CD8 T cells was significantly increased and the proportion of Ly6C^−^ CD8 T cells was markedly lower (Appendix Fig. S1). A decrease in Ly6C^+^ CD8^+^ T cells was also observed after treatment with MSCs; however, Ly6C^−^ CD8^+^ T cells were not significantly affected at day 3 (Fig. 3d, e).

### Effects of MSCs on CD8^+^ T-cell subpopulations

**Fig. 4:**
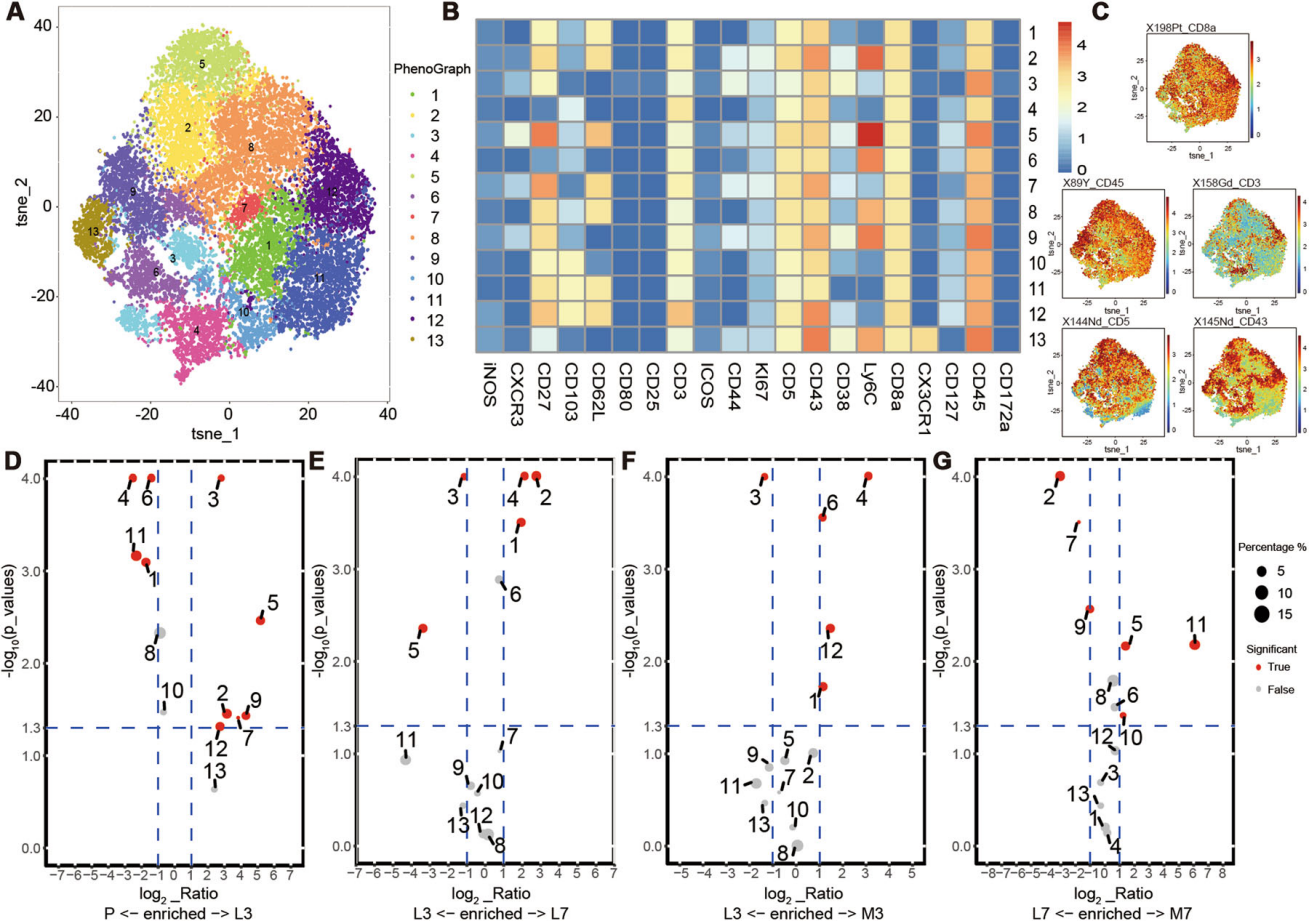
Identification of major CD8^+^ T-cell subsets in ALI treated with MSCs based on mass cytometry. **a** Lung CD8^+^ T-cell cluster analysis based on t-SNE revealed a total of 13 different clusters. **b** Normalized expression of markers in lung CD8^+^ T-cell clusters shown as a heatmap. **c** t-SNE map showing the relative expression levels of the markers. Volcano of differentially abundant clusters between **d** P vs. L3, **e** L3 vs. L7, **f** L3 vs. M3, and **g** L7 vs. M7. P, PBS/PBS group; L3, LPS/PBS group at day 3; L7, LPS/PBS group at day 7; M3, LPS/MSC group at day 3; M7, LPS/MSC group at day 7 (based on unpaired Student’s *t*-test).

The dynamics and distribution of the CD8^+^ T cells were assessed based on t-SNE. The CD8^+^ T cells displayed distinct dynamics and distribution trends during the disease process (Appendix Fig. S2). Expression levels of CD3, CD5, CD43, CD8a, and CD45 were high in all CD8^+^ T cells (Fig. 4c). The levels of the other markers are shown in Appendix Fig. S2.

The extracted CD8^+^ T cells were further analyzed and subdivided into 13 clusters (Fig. 4a). The normalized expression levels of the 13 clusters are shown as a heatmap (Fig. 4b). Cluster abundance volcano plots were used to analyze the dynamic changes in different CD8^+^ T-cell subsets during the course of ALI. On day 3, clusters 1, 4, 6, and 11 were mainly enriched in the WT mice, whereas clusters 3 and 5 were predominant after treatment with LPS (Fig. 4d, e). Furthermore, CXCR3 was expressed in cluster 3 and at a higher level in cluster 5 (Fig. 4b). Clusters 1, 4, and 11 expressed Ly6C at a low level, consistent with the predominance of Ly6C^−^ CD8^+^ T cells in the lungs of WT mice. As ALI progressed, cluster 2 was predominant in the LPS/PBS group at day 7 (Fig. 4e, g). After administration of MSCs, cluster 4 became more enriched at day 3 in the LPS/MSC group, with a low level of CD27, which is a co-stimulatory receptor that promotes immune activation^27^ (Fig. 4f). Cluster 11 was enriched in the lungs of ALI mice treated with MSCs compared with the LPS/PBS group at day 7 (Fig. 4g). Clusters 2, 3, 5, and 6 expressed CD44 and Ly6C at high levels, indicating an activated phenotype (Fig. 4b). However, clusters 1, 4, and 11 expressed CD44 and Ly6C at low levels, indicating a naive phenotype (Fig. 4b). Furthermore, clusters 4 and 11 were positive for CD103, which is a marker expressed on tissue-resident cells (Fig. 4b).

### Ly6c^+^ Cd8a^+^ T cells exhibited a more activated phenotype based on scRNA-seq

**Fig. 5:**
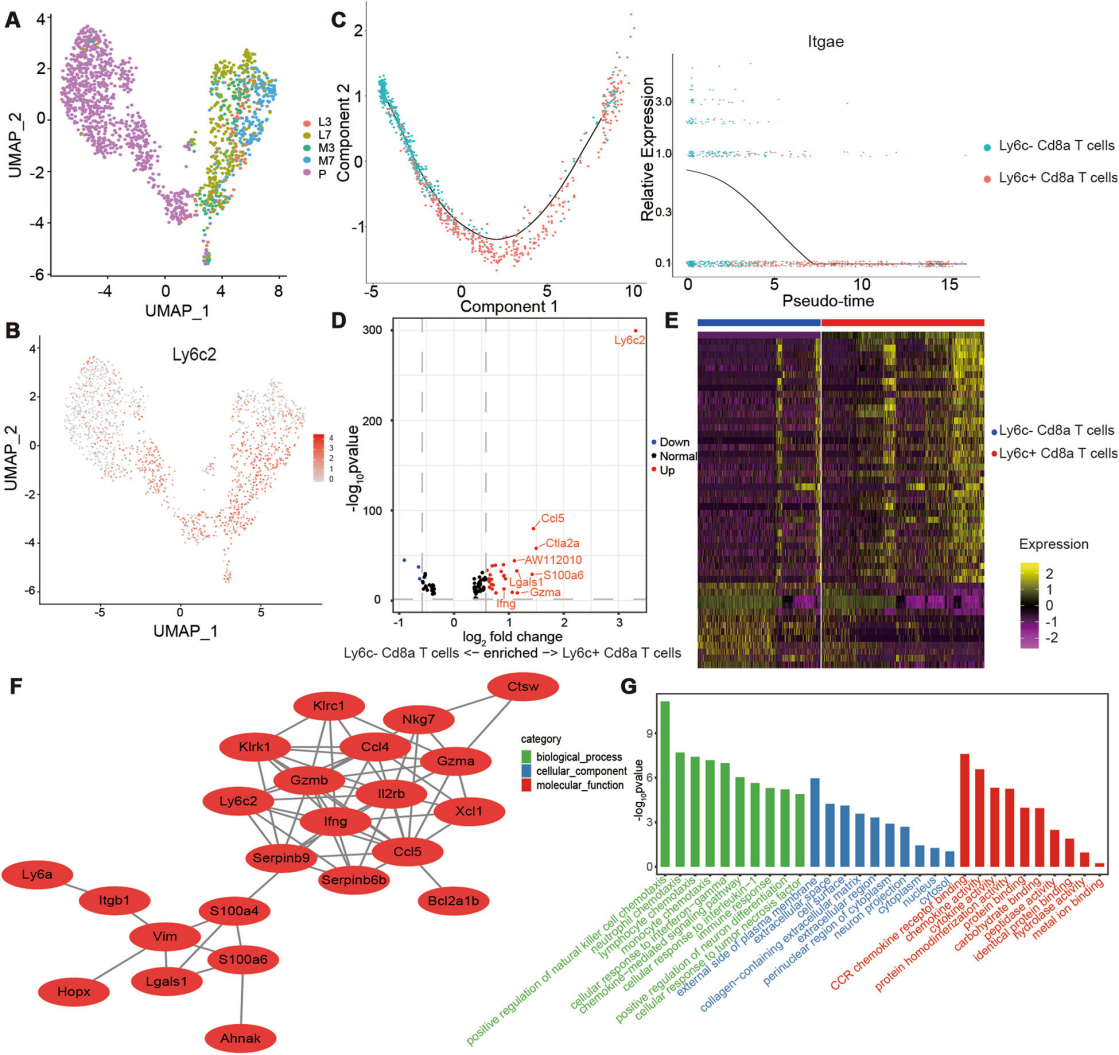
Activated phenotype of Ly6c^+^ Cd8a^+^ T cells based on scRNA-seq. **a** UMAP dimensionality reduction of Cd8a^+^ T cells in the lung. Colors indicate the sample type. **b** Feature plot of the Ly6c2 in lung Cd8a^+^ T cells based on normalized expression. **c** Pseudotime ordering of the Ly6c^−^ and Ly6c^+^ Cd8a^+^ T cells and expression changes of *Itgae*. **d** Volcano plot for DEGs between Ly6c^−^ and Ly6c^+^ Cd8a^+^ T cells. **e** Heatmap of DEGs between Ly6c^−^ and Ly6c^+^ Cd8a^+^ T cells. **f** PPI network of DEGs upregulated in Ly6c^+^ vs. Ly6c^−^ Cd8a^+^ T cells. **g** Top 30 GO terms of DEGs upregulated in Ly6c^+^ vs. Ly6c^−^ Cd8a^+^ T cells. DEGs, *P* < 0.05, fold change > 1.5.

The scRNA-seq profiles were analyzed for in terms of CD8^+^ T-cell expression during ALI. UMAP analysis identified CD8^+^ T cells based on their signature gene Cd8a (Appendix Fig. S3). A two-dimensional UMAP plot showed the differentiation of cells in normal mice vs. mice with ALI (Fig. 5a). The expression pattern of Ly6c2 in Cd8a^+^ cells was similar to the results of mass cytometry analysis, which showed an increased proportion of Ly6C^+^ CD8^+^ T cells after treatment with LPS (Fig. 5a, b).

Pseudotime analysis of Cd8a^+^ T cells isolated from normal lungs showed a trajectory of Ly6c^−^ and Ly6c^+^ Cd8a^+^ T cells, whereas *Itgae* decreased along the trajectory, supporting higher expression of CD103 in Ly6C^−^ CD8^+^ T cells similar to the mass cytometry results (Figs. 4b and 5c). The heatmap showed the genes with significantly different expression patterns between these two subsets, separating them according to their transcriptome characteristics (Fig. 5e).

The Cd8a^+^ T cells were divided into Ly6c^−^ and Ly6c^+^ Cd8a^+^ T cells based on Ly6c2 expression. DEGs in Ly6c^−^ vs. Ly6c^+^ Cd8a^+^ T cells were also illustrated by a volcano plot (Fig. 5d). In addition to Ly6c2, the Ly6c^+^ Cd8a^+^ T cells tended to express higher *Ccl5*, *Gzma*, and *Ifng* levels. All DEGs are shown in Appendix Table S2. Twenty-nine DEGs were imported into PPI networks, including 3 downregulated and 26 upregulated genes. Only 23 of the 29 DEGs were contained in the DEG PPI network (Fig. 5f). The top ten hub genes were ranked using the MCC method, including *Ifng*, *Gzmb*, *Il2rb*, *Ccl5*, *Serpinb9*, *Ly6c2*, *Serpinb6b*, *Klrk1*, *Gzma*, and *Klrc1* (Appendix Table S5). The upregulated genes analyzed using GO enrichment were specifically enriched in many immune response terms, including chemokine and cytokine activity, which influenced the chemotaxis of multiple immune cells (Fig. 5g).

### MSCs mainly inhibited the proinflammatory effects of Ly6c^+^ Cd8a^+^ T cells

**Fig. 6:**
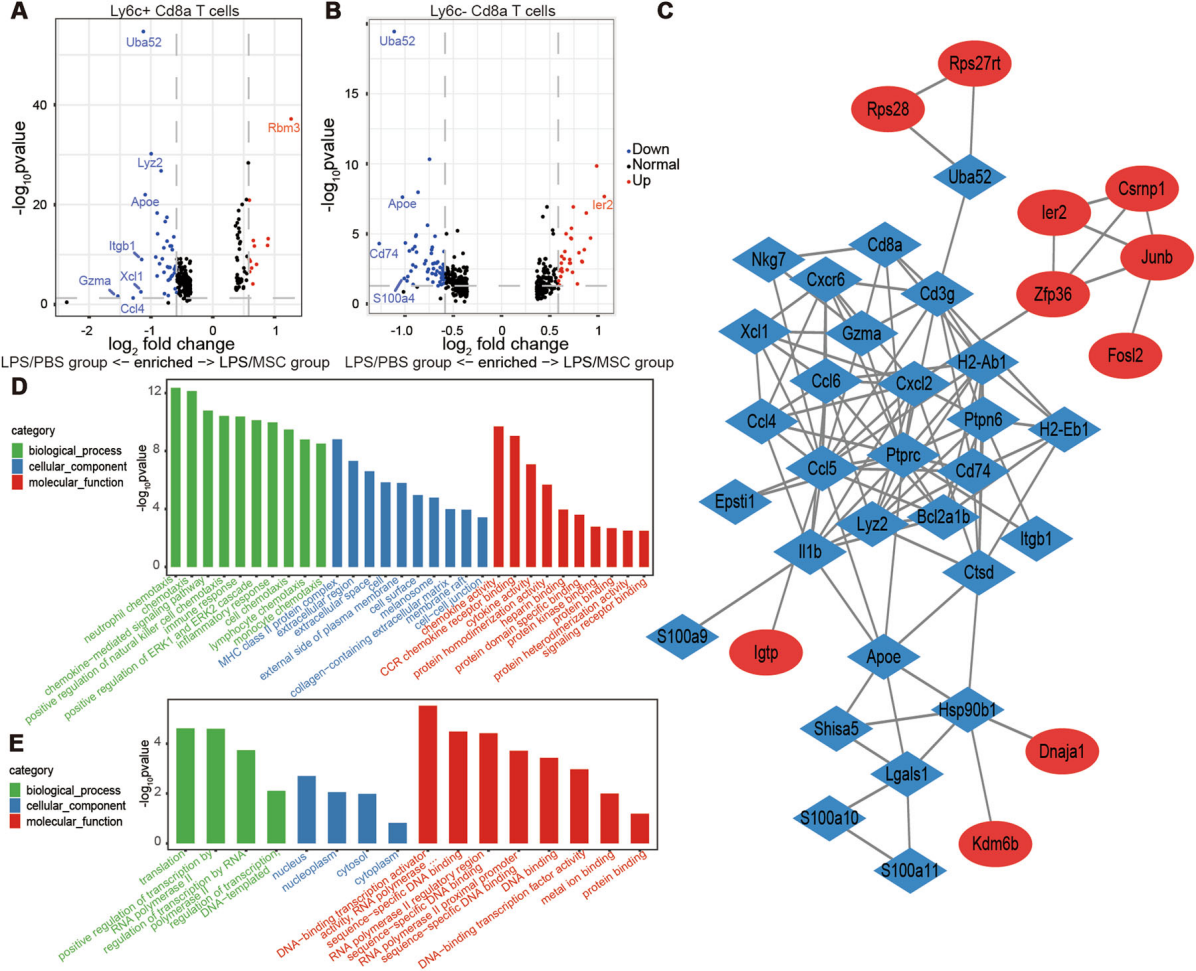
MSCs inhibit the proinflammatory effects of Ly6c^+^ Cd8a^+^ T cells. **a** Volcano plot of DEGs in Ly6c^+^ Cd8a^+^ T cells in the LPS/PBS group vs. LPS/MSC group at day 7. **b** Volcano plot of DEGs in Ly6c^−^ Cd8a^+^ T cells in the LPS/PBS group vs. LPS/MSC group at day 7. Up represents upregulated in the lungs of the LPS/MSC group and Down represents downregulated. **c** PPI network of DEGs in Ly6c^+^ Cd8a^+^ T cells in the LPS/PBS group vs. LPS/MSC group. **d** Top 30 GO terms of DEGs downregulated in Ly6c^+^ Cd8a^+^ T cells in the LPS/PBS group vs. LPS/MSC group. **e** Top 30 GO terms of DEGs upregulated in Ly6c^+^ Cd8a^+^ T cells in the LPS/PBS group vs. LPS/MSC group, DEGs, *P* < 0.05, fold change > 1.5.

To assess the effects of MSCs on the two Cd8a^+^ T subsets, DEGs of Ly6c^−^ and Ly6c^+^ Cd8a^+^ T cells were analyzed in the LPS/PBS and LPS/MSC groups on day 7, respectively. A total of 52 DEGs were found in Ly6c^+^ Cd8a^+^ T cells and there were 94 in Ly6c^−^ Cd8a^+^ T cells (Appendix Tables S3 and S4). Volcano maps of the DEGs showed that the inflammation-related factors, such as *Gzma*, *Ccl4*, and *Xcl1*, were downregulated in Ly6c^+^ Cd8a^+^ T cells after treatment with MSCs. However, these genes were not found in Ly6c^−^ Cd8a^+^ cells (Fig. 6a, b). PPI networks indicated a module that contained downregulated genes in Ly6c^+^ Cd8a^+^ T cells in the LPS/MSC group (Fig. 6c). Many genes were upregulated and downregulated in Ly6c^−^ Cd8a^+^ T cells after treatment with MSCs (Appendix Fig. S3). After treatment with MSCs, the top ten hub genes indicated that more inflammatory-related genes were downregulated in Ly6c^+^ Cd8a^+^ T cells compared with Ly6c^−^ Cd8a^+^ T cells (Appendix Tables S6 and S7). The downregulated genes in the Ly6c^+^ Cd8a^+^ T cells displayed biological process enrichment in immune and inflammatory responses, together with many terms associated with chemotaxis in biological processes and molecular functions after treatment with MSCs (Fig. 6d). However, upregulated and downregulated genes in Ly6c^−^ Cd8a^+^ T cells did not display specific enrichment in inflammatory-related terms, similar to the upregulated genes in Ly6c^+^ Cd8a^+^ T cells after treatment with MSCs (Fig. 6e and Appendix Fig. S3).

## Discussion

ALI causes immune dysfunction, promotes the release of proinflammatory factors, increases the number of white blood cells, and can progress to ARDS. In the present study, an animal model of LPS-induced ALI was used to explore several complex characteristics of the disease in humans. The survival rate and histopathology analyses indicated that lung injury was most severe at day 3 after ALI; the lung gradually returned to normal by day 7 and MSC treatment improved survival. Days 3 and 7 were therefore chosen to represent the initial inflammatory phase and resolution phase, respectively. Progression of lung damage is associated with an increased number of inflammatory cells (predominantly neutrophils) and increased protein levels, as well as increased expression of CCL3, CCL4, CCL5, CXCL1, IFN-γ, TNF-α, and IL6 in BALF. CD8^+^ T-cell infiltration in lung tissue increased as the disease progressed, indicating a contribution to inflammation-mediated tissue injury. Consistent with our results, inhibition of CD8^+^ T cells was shown in previous studies to play an important role in regulating a variety of immune responses, including airway inflammation^28–30^. Claser et al.^31^ also discovered that depletion of CD8^+^ T cells with CD8β antibody can protect the pulmonary epithelium from damage in cases of malaria-associated ALI. Increased infiltration of CD8^+^ T cells exacerbates tissue damage when the disease progresses and inhibition of CD8^+^ T cells can help in the treatment of the diseases.

MSCs have potential as a therapeutic approach for pneumonia due to their immunosuppressive activity. The lower inflammatory response and longer survival rates observed in ALI treated with MSCs support this conclusion. The extensive anti-inflammatory activity of MSCs in the lungs likely promotes the recovery of ALI through a paracrine mechanism by secreting soluble factors. The reduction of these inflammatory factors may result from the decreased inflammatory permeability of the endothelial and epithelial tissues of the BALF in mice treated with MSCs, as evidenced by the lower concentration of BALF proteins in MSC-treated ALI mice compared with the LPS/PBS group. The results of this study showed that infiltration of CD8^+^ T cells was decreased in ALI mice treated with MSCs, in turn indicating that LPS-induced ALI ameliorated by MSCs was associated with a reduction of CD8^+^ T cells. In previous studies, MSCs, and their production of indoleamine 2,3 dioxygenase, were shown to contribute to the induction of apoptosis in activated T cells^32,33^ and high stanniocalcin-2 expression decreased CD8^+^ cytotoxic T cells during MSC-based treatment for allergic contact dermatitis^15^. Taken together, our results indicate that MSCs may help prevent or treat ALI by affecting CD8^+^ T cells.

Ly6C^+^ CD8^+^ T cells are the main CD8^+^ T-cell population during infiltration. Ly6C, a marker of immunological processes such as T-cell activation and augmented effector function, is reportedly expressed in CD8^+^ T cells and is believed to correlate with enhanced cytotoxic activity^20,34–37^. Single-cell pseudotime sorting showed a trajectory between Ly6c^−^ and Ly6c^+^ Cd8a^+^ T cells in WT mice. The gene *Itgae*, which codes for CD103, a member of the integrin family (αEβ7) that interacts with E-cadherin, may aid CD8^+^ T cells in maintaining their position within the lung^38,39^. These data showed that Ly6C^−^ CD8^+^ T cells have higher CD103 expression, thus representing tissue-resident cells. However, DEGs upregulated in Ly6c^+^ vs. Ly6c^−^ Cd8a^+^ T cells showed a significant association with immunological and inflammatory responses, as reflected in the expression levels of typical cytokines, chemokines, and cytotoxic factors, such as *Ifng* (encoding IFN-γ), *Ccl5* (encoding CCL5), *Gzma* (encoding granzyme GZMA), and *Gzmb* (encoding GZMB). Previous research showed GZMB may be involved in endothelial intimal rupture by reducing the expression of tight junction proteins^31^. Kusaka et al.^19^ also reported that Ly6C^+^ CD8^+^ T cells were a major source of IFN-γ in CD3^+^ cells and played an important role during the acute phase of infection. These data indicate that Ly6C^+^ CD8^+^ T cells have an activated phenotype and contribute to ALI progression. MSC-ameliorated lung injury was associated with a reduction of Ly6C^+^ CD8^+^ T cells.

The inflammatory response is complicated; therefore, researching the effects of MSCs on the immune reaction (e.g., on the activity of CD8^+^ T cells) is important. In-depth profiling using mass cytometry and scRNA-seq showed that CD8^+^ cell expression in lungs treated with MSCs differs from that in non-treated lung cells. Clusters 3 and 5, expressing a high CXCR3 level, were the predominant CD8^+^ T cells in ALI at day 3. CXCR3 is an important chemokine receptor in the migration of CD8^+^ T cells to multiple tissues, especially in the context of inflammation and infection^40^. CXCR3 expression significantly amplifies the cytotoxic potential of CD8^+^ T cells and the ability to produce IFN-γ^41^. Excessive production of IFN-γ due to increased infiltration of CD8^+^ T cells in the lungs is thought to lead to ALI^42^. Knockout of the *CXCR3* gene has been shown to attenuate ALI in mice with acute pancreatitis^43^, which is in agreement with our results. CD8^+^ T cells in the lungs expressed low CXCR3 level after treatment with MSCs. Ly6C^+^ CD8^+^ T cells with high CXCR3 expression play a pivotal role in the pathogenesis and progression of ALI. MSCs can alleviate lung injury by suppressing CXCR3 expression in CD8^+^ T cells.

In this study, further characterization of the expression profile of Cd8a^+^ T cells using scRNA-seq showed that the downregulated DEGs in Ly6c^+^ Cd8a^+^ T cells of mice treated with MSCs were specifically enriched in GO terms for immune cell chemotaxis (especially neutrophil chemotaxis). The process by which MSCs alleviate ALI may be associated with a reduction in the inflammatory cytokines released by Ly6c^+^ Cd8a^+^ T cells. Downregulated DEGs, such as *Ccl4*, *Ccl5*, *Ccl6*, *Cxcl2*, and *Xcl1*, were involved in the chemotaxis of neutrophils, corresponding to increased infiltration of neutrophils in BALF. Neutrophils play an important role in ALI^44^. Activated neutrophils enhance their chemotaxis and adhesion to endothelial cells, and migrate into the lung interstitium, which restricts the repair of lung tissues^45^. Inhibiting Fms-like tyrosine kinase 3-mediated activation of neutrophils was shown to alleviate LPS-induced ALI^46^. The results of the present study indicated that MSCs may alleviate the lung damage due to infiltrating neutrophils by inhibiting chemotaxis in Ly6C^+^ CD8^+^ T cells.

In conclusion, our results indicated that LPS-induced models in which concurrent application of MSCs and decreased infiltration of Ly6C^+^ CD8^+^ T cells helped alleviate ALI. Most previous studies focused on the effects of innate immune cells in ALI. The immunoregulatory effect of MSCs in the treatment of ALI is not only applicable to a single immune cell subpopulation but also to the entire immune system as a regulatory network. Our data showed that MSCs correlated with the decreased number of Ly6C^+^ CD8^+^ T cells, as well as inhibition of their function. The results of this study improve our understanding of the immune mechanism underlying MSC-mediated improvement in ALI and may provide a novel therapeutic method to modulate the response to ALI.

## Supplementary information

Supplementary Data

Supplementary Figure Legends

Supplementary Table Legends

supplementary Figure S1

supplementary Figure S2

supplementary Figure S3

## Data Availability

All custom code is available from the corresponding author upon reasonable request.
