# Supplementary material for: Mesenchymal stem cells alleviate LPS-induced acute lung injury by inhibiting the proinflammatory function of Ly6C+ CD8+ T cells
Source: Cell Death Dis. 2020 Oct 6;11(10):829. doi: 10.1038/s41419-020-03036-1 (PMC7538431; doi:10.1038/s41419-020-03036-1)
Supplement: Supplementary file 1 — Supplementary Data [file 41419_2020_3036_MOESM1_ESM.docx]

**Appendix data**

**Mesenchymal stem cells alleviate LPS-induced acute lung injury by the inhibiting proinflammatory function of Ly6C^+^ CD8^+^ T cells**

Jiaqi Zhu^1,2^, Bing Feng^1,2^, Yanping Xu^1,2^, Wenyi Chen^1,2^, Xinyu Sheng^1,2^, Xudong Feng^1,2^, Xiaowei Shi^1^, Jingqi Liu^1^, Qiaoling Pan^1,2^, Jinfeng Yang, Jiong Yu^1,2^, Lanjuan Li^1,2^, Hongcui Cao†^1,2,3^

1 State Key Laboratory for the Diagnosis and Treatment of Infectious Diseases, the First Affiliated Hospital, College of Medicine, Zhejiang University, 79 Qingchun Rd., Hangzhou City 310003, China

2 National Clinical Research Center for Infectious Diseases, 79 Qingchun Rd., Hangzhou City 310003, China

3 Zhejiang Provincial Key Laboratory for Diagnosis and Treatment of Aging and Physic-chemical Injury Diseases, 79 Qingchun Rd, Hangzhou City 310003, China.

**†Corresponding author:**

Hongcui Cao

State Key Laboratory for the Diagnosis and Treatment of Infectious Diseases, the First Affiliated Hospital, College of Medicine, Zhejiang University, 79 Qingchun Rd., Hangzhou City 310003, China. Tel: 86-571-87236451; Fax: 86-571-87236459

E-mail: hccao@zju.edu.cn

**Appendix method**

***Isolation and culture of mouse MSCs***

The isolation and culture of mouse bone marrow MSCs were performed as previously described.^1^ C57BL/6 male mice (2–3 weeks of age) were sacrificed. Humeri, tibiae, and femurs were collected, and the marrow was thoroughly flushed out with 3 mL α-minimal essential medium (GE Healthcare Life Sciences, Logan, UT, USA). The bones were cut into approximately 1–2-mm^3^ pieces and digested with 2 mg/mL collagenase II digestion solution (Life Technologies, Grand Island, NY, USA) at 37°C for 1.5 h. After digestion, the bone chips were incubated in MSC special complete medium (Cyagen Biosciences, Guangzhou, China) at 37°C in a 5% CO_2_ incubator (Thermo Fisher Scientific Inc., Waltham, MA, USA). Non-adherent cells were removed by replacing the complete medium on day 3. On day 5, adherent cells were harvested with 0.25% trypsin-EDTA (Invitrogen, Carlsbad, CA, USA) and incubated in fresh MSC special complete medium at 37°C in a 5% CO_2_ incubator. MSCs at passage 3 were used for subsequent experiments.

The isolated MSCs were identified through the induction of osteocytes, adipocytes and based on the expression of specific markers.^2^ MSCs were cultured in osteogenic differentiation medium (Cyagen Biosciences) and adipogenic differentiation medium (Cyagen Biosciences) with appropriate supplements. In addition, the cultures were stained after 4 weeks with Alizarin Red S (Cyagen Biosciences) to examine the induction of osteocytes, and with Oil Red O (Cyagen Biosciences) for adipogenic induction. Primary staining was performed for phenotypic analysis of MSCs using monoclonal antibodies including anti-APC-CD29 (HMβ1-1; Biolegend®, San Diego, CA, USA), anti-APCCy7-CD11b (M1/70; Biolegend), anti-APCCy7-MCH Ⅱ-1A (M5/114.15.2; Biolegend), anti-PE-CD44 (IM7; Biolegend), anti-PE-SCA-1 (D7; Biolegend), anti-PECy7-CD31 (390; Biolegend), anti-PerCP-CD86 (GL-1; Biolegend), and anti-PECy7-CD45 (30-F11; Biolegend). MSCs were incubated with the above-mentioned antibodies and corresponding isotype controls (Biolegend) for 30 min at 4℃ in the dark. After washing twice with PBS (Hangzhou Gino Bio-pharmaceutical Technology Co. Ltd., Zhejiang, China) containing 0.5% bovine serum albumin (Sangon Biotech, Shanghai, China), the MSCs were analyzed using flow cytometry (BeamCyte-1026; Beamdiag, Changzhou, China).

**Appendix references**

1. Zhu, H. *et al.* A protocol for isolation and culture of mesenchymal stem cells from mouse compact bone. *Nat Protoc* **5**, 550-560 (2010).
2. Liu, J. *et al.* Immunomodulatory effect of mesenchymal stem cells in chemical-induced liver injury: a high-dimensional analysis. *Stem Cell Res Ther* **10**, 262 (2019).

**Appendix Table**

**Appendix Table S1: List of 23 metal isotope-tagged antibodies for mass cytometry**

| Supplement Table S1: metal isotope-labeled antibodies for mass cytometry | | | |
| --- | --- | --- | --- |
| marker | label | clone | company |
| CD45 | 89Y | 30-F11 | Fluidigm |
| CD44 | 113In | IM7 | Biolegend |
| CD19 | 115In | 6D5 | Biolegend |
| CD5 | 144Nd | 53-7.3 | Biolegend |
| CD43 | 145Nd | S11 | Biolegend |
| CD38 | 146Nd | 90 | Biolegend |
| Ly6C | 148Nd | HK1.4 | Biolegend |
| CX3CR1 | 149Sm | SA011F11 | Biolegend |
| CD62L | 151Eu | MEL14 | Biolegend |
| TCR γ/δ | 153Eu | GL-3 | homemade |
| anti-APC |  | APC003 | Biolegend |
| CD80 | 155Gd | 16-10A1 | Biolegend |
| CD25 | 157Gd | 3C7 | Biolegend |
| CD3 | 158Gd | 145.2C11 | Biolegend |
| CXCR3 | 162Dy | CXCR3-173 | Biolegend |
| CD27 | 163Dy | LG.3A10 | Biolegend |
| CD103 | 164Dy | 2E7 | Biolegend |
| ICOS | 165Ho | C398.4A | Biolegend |
| CD127 | 169Tm | A7R34 | Biolegend |
| CD172a | 173Yb | P84 | Biolegend |
| CD4 | 197gd | RM4-5 | Biolegend |
| CD8a | 198pt | 53-6.7 | Biolegend |
| intracellular marker | |  |  |
| Ki67 | 139La | SolA15 | eBioscience |
| iNOS | 161Dy | CXNFT | Fluidigm |

**Appendix Table S2: DEGs between Ly6c^-^ Cd8a^+^ T cells and Ly6c^+^ Cd8a^+^ T cells**

| (Ly6c- Cd8a+ T cells) versus (Ly6c+ Cd8a+ T cells)-diff-pval-0.05-FC-1.5 | | | | | |
| --- | --- | --- | --- | --- | --- |
| GeneID | pvalue | padj | FoldChange | log2FoldChange | up_down |
| AW112010 | 9.41E-45 | 2.92E-40 | 2.137728 | 1.096079 | Up |
| Ahnak | 2.71E-33 | 8.41E-29 | 1.807079 | 0.85366 | Up |
| Bcl2a1b | 4.50E-18 | 1.40E-13 | 1.637563 | 0.711551 | Up |
| Ccl4 | 1.17E-15 | 3.63E-11 | 1.558668 | 0.640313 | Up |
| Ccl5 | 2.25E-80 | 6.99E-76 | 2.719488 | 1.443335 | Up |
| Ctla2a | 1.20E-58 | 3.72E-54 | 2.815126 | 1.4932 | Up |
| Ctsw | 4.30E-39 | 1.33E-34 | 1.617149 | 0.693452 | Up |
| Gm42418 | 1.06E-24 | 3.29E-20 | 0.645887 | -0.63065 | Down |
| Gzma | 2.29E-09 | 7.13E-05 | 2.220627 | 1.150967 | Up |
| Gzmb | 1.90E-09 | 5.91E-05 | 1.692239 | 0.758934 | Up |
| Hopx | 5.61E-34 | 1.74E-29 | 1.512595 | 0.597026 | Up |
| Ifng | 8.07E-10 | 2.51E-05 | 2.076651 | 1.054258 | Up |
| Il2rb | 8.94E-40 | 2.78E-35 | 1.682339 | 0.750468 | Up |
| Itgb1 | 8.47E-29 | 2.63E-24 | 1.873726 | 0.90591 | Up |
| Klrc1 | 1.90E-24 | 5.91E-20 | 1.598132 | 0.676387 | Up |
| Klrk1 | 6.86E-19 | 2.13E-14 | 1.595127 | 0.673671 | Up |
| Lef1 | 6.17E-38 | 1.91E-33 | 0.635963 | -0.65298 | Down |
| Lgals1 | 8.07E-34 | 2.50E-29 | 2.204229 | 1.140274 | Up |
| Ly6a | 1.05E-24 | 3.26E-20 | 1.907493 | 0.931678 | Up |
| Ly6c2 | 0 | 0 | 9.904071 | 3.308022 | Up |
| Nkg7 | 1.28E-40 | 3.99E-36 | 1.862433 | 0.897189 | Up |
| Plac8 | 4.81E-28 | 1.49E-23 | 1.866184 | 0.900091 | Up |
| Rgcc | 1.35E-45 | 4.19E-41 | 0.531551 | -0.91172 | Down |
| S100a4 | 1.52E-13 | 4.71E-09 | 1.875025 | 0.90691 | Up |
| S100a6 | 1.28E-29 | 3.97E-25 | 2.676197 | 1.420185 | Up |
| Serpinb6b | 1.87E-29 | 5.80E-25 | 1.574769 | 0.65514 | Up |
| Serpinb9 | 3.97E-25 | 1.23E-20 | 1.54981 | 0.632091 | Up |
| Vim | 1.27E-21 | 3.94E-17 | 1.554357 | 0.636318 | Up |
| Xcl1 | 1.32E-13 | 4.11E-09 | 1.614969 | 0.691507 | Up |

**Appendix Table S3: DEGs of Ly6c^+^ Cd8a^+^ T cells between the LPS/PBS group and the LPS/MSC group at day 7**

| Ly6c+ Cd8a+ T cells_L7-vs-M7-diff-pval-0.05-FC-1.5 | | | | | |
| --- | --- | --- | --- | --- | --- |
| GeneID | pvalue | padj | FoldChange | log2FoldChange | up_down |
| AW112010 | 4.24E-08 | 0.001318 | 0.608079 | -0.71767 | Down |
| Apoe | 1.11E-22 | 3.46E-18 | 0.467392 | -1.09729 | Down |
| Bcl2a1b | 0.00426 | 1 | 0.666029 | -0.58634 | Down |
| Ccl4 | 0.04726 | 1 | 0.408709 | -1.29085 | Down |
| Ccl5 | 0.018636 | 1 | 0.606068 | -0.72245 | Down |
| Ccl6 | 9.11E-08 | 0.002828 | 0.649309 | -0.62302 | Down |
| Cd3g | 5.20E-19 | 1.62E-14 | 0.534219 | -0.9045 | Down |
| Cd74 | 6.95E-09 | 0.000216 | 0.549085 | -0.8649 | Down |
| Cd8a | 2.52E-14 | 7.83E-10 | 0.644243 | -0.63432 | Down |
| Csrnp1 | 6.54E-14 | 2.03E-09 | 1.855422 | 0.891747 | Up |
| Ctsd | 1.91E-12 | 5.92E-08 | 0.61802 | -0.69427 | Down |
| Cxcl2 | 7.63E-15 | 2.37E-10 | 0.537366 | -0.89602 | Down |
| Cxcr6 | 1.57E-05 | 0.48716 | 0.606006 | -0.7226 | Down |
| Dnaja1 | 1.51E-09 | 4.68E-05 | 1.51038 | 0.594912 | Up |
| Epsti1 | 2.75E-08 | 0.000853 | 0.600171 | -0.73655 | Down |
| Fosl2 | 2.58E-09 | 8.01E-05 | 1.518392 | 0.602544 | Up |
| Gm12840 | 0.000801 | 1 | 0.654357 | -0.61185 | Down |
| Gzma | 0.023728 | 1 | 0.344133 | -1.53896 | Down |
| H2-Ab1 | 3.22E-10 | 1.00E-05 | 0.525928 | -0.92706 | Down |
| H2-Eb1 | 1.69E-06 | 0.0524 | 0.634631 | -0.65601 | Down |
| Hmgb2 | 3.96E-07 | 0.012299 | 0.577217 | -0.79281 | Down |
| Hnrnpc | 1.90E-12 | 5.91E-08 | 0.627966 | -0.67124 | Down |
| Hsp90b1 | 7.89E-06 | 0.245008 | 0.616938 | -0.6968 | Down |
| Ier2 | 9.40E-09 | 0.000292 | 1.628045 | 0.70314 | Up |
| Ifi27l2a | 0.006594 | 1 | 0.580107 | -0.78561 | Down |
| Igtp | 1.65E-12 | 5.13E-08 | 1.842154 | 0.881394 | Up |
| Il1b | 4.26E-07 | 0.013234 | 0.650076 | -0.62132 | Down |
| Itgb1 | 1.12E-09 | 3.47E-05 | 0.449564 | -1.1534 | Down |
| Junb | 1.80E-12 | 5.58E-08 | 1.591549 | 0.670431 | Up |
| Kdm6b | 2.41E-07 | 0.007472 | 1.507084 | 0.59176 | Up |
| Lgals1 | 2.21E-06 | 0.06873 | 0.534287 | -0.90431 | Down |
| Lyz2 | 6.28E-31 | 1.95E-26 | 0.499697 | -1.00087 | Down |
| Mat2a | 5.56E-08 | 0.001725 | 1.542206 | 0.624996 | Up |
| Myl6 | 1.83E-13 | 5.68E-09 | 0.597311 | -0.74345 | Down |
| Nkg7 | 2.59E-06 | 0.080524 | 0.626832 | -0.67385 | Down |
| Ptpn6 | 3.92E-08 | 0.001217 | 0.617003 | -0.69665 | Down |
| Ptprc | 3.54E-18 | 1.10E-13 | 0.596658 | -0.74502 | Down |
| Rbm3 | 6.31E-38 | 1.96E-33 | 2.400618 | 1.263406 | Up |
| Rps27rt | 1.60E-13 | 4.97E-09 | 1.57158 | 0.652216 | Up |
| Rps28 | 1.38E-21 | 4.30E-17 | 1.504222 | 0.589018 | Up |
| S100a10 | 6.54E-10 | 2.03E-05 | 0.566256 | -0.82047 | Down |
| S100a11 | 4.44E-08 | 0.00138 | 0.653705 | -0.61329 | Down |
| S100a9 | 4.90E-12 | 1.52E-07 | 0.576214 | -0.79532 | Down |
| Serf2 | 2.31E-17 | 7.18E-13 | 0.585418 | -0.77246 | Down |
| Sh3bgrl3 | 3.74E-08 | 0.001162 | 0.662603 | -0.59378 | Down |
| Shisa5 | 1.22E-11 | 3.78E-07 | 0.633629 | -0.65829 | Down |
| Themis | 2.34E-08 | 0.000726 | 0.658083 | -0.60366 | Down |
| Tmsb4x | 1.55E-27 | 4.80E-23 | 0.558603 | -0.84011 | Down |
| Uba52 | 2.29E-55 | 7.10E-51 | 0.458246 | -1.12581 | Down |
| Wdr89 | 9.75E-10 | 3.03E-05 | 1.500382 | 0.58533 | Up |
| Xcl1 | 0.002745 | 1 | 0.446068 | -1.16466 | Down |
| Zfp36 | 8.16E-05 | 1 | 1.563793 | 0.64505 | Up |

**Appendix Table S4: DEGs of Ly6c^-^ Cd8a^+^ T cells between the LPS/PBS group and the LPS/MSC group at day 7**

| Ly6c- Cd8a+ T cells_L7-vs-M7-diff-pval-0.05-FC-1.5 | | | | |  |
| --- | --- | --- | --- | --- | --- |
| GeneID | pvalue | padj | FoldChange | log2FoldChange | up_down |
| AW112010 | 0.01151 | 1 | 0.509187 | -0.97373 | Down |
| Ahnak | 0.016397 | 1 | 0.587155 | -0.76819 | Down |
| Anxa2 | 0.008463 | 1 | 0.623108 | -0.68245 | Down |
| Apoe | 2.50E-08 | 0.000778 | 0.490025 | -1.02907 | Down |
| B4galnt1 | 0.00824 | 1 | 0.649935 | -0.62163 | Down |
| Batf | 0.000612 | 1 | 1.565266 | 0.646408 | Up |
| Bcl2 | 0.000195 | 1 | 0.541869 | -0.88398 | Down |
| Bcl2a1d | 0.028 | 1 | 0.641118 | -0.64134 | Down |
| Cd28 | 0.007076 | 1 | 0.605972 | -0.72268 | Down |
| Cd74 | 5.21E-05 | 1 | 0.414471 | -1.27066 | Down |
| Cox5a | 0.018311 | 1 | 0.651102 | -0.61904 | Down |
| Cox7c | 0.001274 | 1 | 0.625656 | -0.67656 | Down |
| Csrnp1 | 0.000216 | 1 | 1.741245 | 0.80012 | Up |
| Ctla2a | 0.023209 | 1 | 0.506401 | -0.98165 | Down |
| Ctsw | 0.013884 | 1 | 0.648489 | -0.62485 | Down |
| Cxcl2 | 8.96E-05 | 1 | 0.606619 | -0.72114 | Down |
| Cyld | 0.033367 | 1 | 0.66002 | -0.59942 | Down |
| Dgat1 | 0.042841 | 1 | 0.649252 | -0.62315 | Down |
| Dnajb1 | 3.93E-06 | 0.122098 | 1.648451 | 0.721111 | Up |
| Dnajc15 | 0.007845 | 1 | 0.620569 | -0.68834 | Down |
| Dock2 | 0.004336 | 1 | 0.655708 | -0.60887 | Down |
| Dtx1 | 0.001229 | 1 | 1.534435 | 0.617708 | Up |
| Edf1 | 0.015041 | 1 | 0.661282 | -0.59666 | Down |
| Epsti1 | 0.004928 | 1 | 0.586451 | -0.76992 | Down |
| Fosb | 0.003383 | 1 | 0.590637 | -0.75966 | Down |
| Frmd4b | 2.16E-05 | 0.670435 | 1.648271 | 0.720954 | Up |
| Gadd45g | 0.00538 | 1 | 1.661413 | 0.732411 | Up |
| Gimap4 | 0.000172 | 1 | 0.514991 | -0.95738 | Down |
| Gm12840 | 0.029028 | 1 | 0.609847 | -0.71348 | Down |
| H2-Aa | 0.000847 | 1 | 0.57924 | -0.78777 | Down |
| H2-Eb1 | 0.019399 | 1 | 0.659341 | -0.6009 | Down |
| Hist1h1c | 0.005468 | 1 | 0.66518 | -0.58818 | Down |
| Hnrnpc | 1.21E-05 | 0.376895 | 0.540823 | -0.88677 | Down |
| Hsbp1 | 0.00134 | 1 | 0.609597 | -0.71407 | Down |
| Hsp90aa1 | 0.003134 | 1 | 1.536946 | 0.620067 | Up |
| Icam1 | 0.038556 | 1 | 1.72152 | 0.783683 | Up |
| Ier2 | 2.27E-08 | 0.000705 | 2.087452 | 1.061743 | Up |
| Ifi203 | 0.043368 | 1 | 0.645963 | -0.63048 | Down |
| Ifi27l2a | 0.001718 | 1 | 0.527642 | -0.92237 | Down |
| Ifngr1 | 0.004679 | 1 | 0.627707 | -0.67184 | Down |
| Il1b | 0.002976 | 1 | 0.61204 | -0.7083 | Down |
| Il7r | 0.001287 | 1 | 0.617966 | -0.6944 | Down |
| Irf1 | 0.000921 | 1 | 1.77751 | 0.829858 | Up |
| Itgb1 | 0.002321 | 1 | 0.539084 | -0.89142 | Down |
| Junb | 3.26E-07 | 0.010133 | 1.833993 | 0.874988 | Up |
| Krtcap2 | 0.016964 | 1 | 0.665949 | -0.58652 | Down |
| Ldha | 0.001208 | 1 | 1.628084 | 0.703175 | Up |
| Lmo4 | 0.001307 | 1 | 1.636985 | 0.711041 | Up |
| Ltb | 0.001371 | 1 | 0.57452 | -0.79957 | Down |
| Lyz2 | 1.11E-08 | 0.000345 | 0.547784 | -0.86832 | Down |
| Maf | 0.003793 | 1 | 1.59896 | 0.677134 | Up |
| Magt1 | 0.006842 | 1 | 0.663713 | -0.59137 | Down |
| Morf4l1 | 1.51E-05 | 0.470365 | 0.619357 | -0.69116 | Down |
| Mrps15 | 0.006169 | 1 | 0.640945 | -0.64173 | Down |
| Mtpn | 0.01402 | 1 | 0.662901 | -0.59313 | Down |
| Myl6 | 0.00225 | 1 | 0.66053 | -0.5983 | Down |
| Ncor1 | 0.005827 | 1 | 0.657783 | -0.60432 | Down |
| Ndufab1 | 0.002601 | 1 | 0.642391 | -0.63848 | Down |
| Nfkbia | 0.001059 | 1 | 1.78352 | 0.834727 | Up |
| Nfkbid | 0.047222 | 1 | 1.514314 | 0.598664 | Up |
| Nufip2 | 0.004171 | 1 | 1.508543 | 0.593156 | Up |
| Pkn2 | 0.00774 | 1 | 0.664913 | -0.58876 | Down |
| Pla2g16 | 1.55E-05 | 0.480185 | 0.538883 | -0.89196 | Down |
| Ppp1ca | 0.000142 | 1 | 0.604405 | -0.72641 | Down |
| Ptp4a2 | 0.01304 | 1 | 0.65406 | -0.61251 | Down |
| Rbm3 | 1.48E-10 | 4.60E-06 | 1.977038 | 0.98334 | Up |
| Rgs1 | 0.040824 | 1 | 0.529005 | -0.91865 | Down |
| Rinl | 0.010113 | 1 | 0.656659 | -0.60678 | Down |
| Rps27rt | 1.21E-07 | 0.003749 | 1.671677 | 0.741296 | Up |
| Rrp1 | 0.000189 | 1 | 1.572401 | 0.652969 | Up |
| S100a10 | 4.61E-05 | 1 | 0.5002 | -0.99942 | Down |
| S100a11 | 0.003216 | 1 | 0.653415 | -0.61393 | Down |
| S100a4 | 0.016079 | 1 | 0.496272 | -1.0108 | Down |
| S100a6 | 0.004829 | 1 | 0.520702 | -0.94147 | Down |
| S100a9 | 8.01E-05 | 1 | 0.53526 | -0.90169 | Down |
| Saa3 | 1.93E-05 | 0.598432 | 1.641941 | 0.715402 | Up |
| Sbno2 | 0.000421 | 1 | 1.537731 | 0.620803 | Up |
| Serf2 | 0.000188 | 1 | 0.660271 | -0.59887 | Down |
| Shisa5 | 0.001038 | 1 | 0.631541 | -0.66305 | Down |
| Slco3a1 | 0.00148 | 1 | 0.656731 | -0.60662 | Down |
| Socs1 | 1.99E-05 | 0.619199 | 1.891392 | 0.919449 | Up |
| Socs3 | 0.00068 | 1 | 1.629575 | 0.704495 | Up |
| Stat4 | 0.049832 | 1 | 0.633076 | -0.65955 | Down |
| Tgif1 | 0.011732 | 1 | 1.52717 | 0.610861 | Up |
| Tmsb4x | 4.94E-11 | 1.53E-06 | 0.595335 | -0.74823 | Down |
| Tomm7 | 0.000238 | 1 | 0.602756 | -0.73035 | Down |
| Tra2a | 0.022278 | 1 | 1.504176 | 0.588973 | Up |
| Trnp1 | 1.02E-05 | 0.316789 | 1.586573 | 0.665914 | Up |
| Txnip | 0.000124 | 1 | 0.527576 | -0.92255 | Down |
| Uba52 | 3.53E-20 | 1.10E-15 | 0.461794 | -1.11468 | Down |
| Wbp4 | 0.005048 | 1 | 1.541454 | 0.624292 | Up |
| Zcchc7 | 0.007774 | 1 | 0.651023 | -0.61922 | Down |
| Zfp36 | 0.000136 | 1 | 1.807756 | 0.8542 | Up |
| mt-Nd4l | 2.49E-06 | 0.077208 | 0.586415 | -0.77 | Down |

**Appendix Table S5: Top 10 of DEGs between Ly6c^-^ Cd8a^+^ T cells and Ly6c^+^ Cd8a^+^ T cells**

| (Ly6c- Cd8a+ T cells) versus (Ly6c+ Cd8a+ T cells)-diff: Top 10 in network string_interactions.tsv ranked by MCC method | | |
| --- | --- | --- |
| Rank | Name | Score |
| 1 | Ifng | 1159 |
| 2 | Gzmb | 1152 |
| 3 | Il2rb | 1128 |
| 4 | Ccl5 | 1063 |
| 5 | Serpinb9 | 840 |
| 6 | Ly6c2 | 744 |
| 7 | Serpinb6b | 720 |
| 8 | Klrk1 | 384 |
| 9 | Gzma | 290 |
| 10 | Klrc1 | 120 |

**Appendix Table S6: Top 10 of DEGs in Ly6c^+^ Cd8a^+^ T cells between the LPS/PBS group and the LPS/MSC group at day 7**

| Ly6c+ Cd8a+ T cells_L7-vs-M7: Top 10 in network string_interactions.tsv ranked by MCC method | | |
| --- | --- | --- |
| Rank | Name | Score |
| 1 | Ccl5 | 281 |
| 2 | Cxcl2 | 217 |
| 3 | Ccl4 | 192 |
| 4 | Ptprc | 184 |
| 5 | Ccl6 | 174 |
| 5 | Cxcr6 | 174 |
| 7 | Xcl1 | 126 |
| 7 | H2-Ab1 | 126 |
| 9 | Il1b | 91 |
| 10 | Lyz2 | 84 |

**Appendix Table S7: Top 10 of DEGs in Ly6c^-^ Cd8a^+^ T cells between the LPS/PBS group and the LPS/MSC group at day 7**

| Ly6c- Cd8a+ T cells_L7-vs-M7: Top 10 in network string_interactions.tsv ranked by MCC method | | |
| --- | --- | --- |
| Rank | Name | Score |
| 1 | Socs3 | 181 |
| 2 | Il1b | 157 |
| 3 | Irf1 | 156 |
| 4 | Icam1 | 140 |
| 5 | Cxcl2 | 136 |
| 6 | Nfkbia | 125 |
| 7 | Anxa2 | 51 |
| 8 | S100a6 | 48 |
| 8 | S100a10 | 48 |
| 8 | S100a11 | 48 |
