# Supplementary material for: Mesenchymal stem cells alleviate LPS-induced acute lung injury by inhibiting the proinflammatory function of Ly6C+ CD8+ T cells
Source: Cell Death Dis. 2020 Oct 6;11(10):829. doi: 10.1038/s41419-020-03036-1 (PMC7538431; doi:10.1038/s41419-020-03036-1)
Supplement: Supplementary file 2 — Supplementary Figure Legends [file 41419_2020_3036_MOESM2_ESM.docx]

**Appendix figure legends**

**Appendix Figure S1.** **Dynamic changes in the composition of Ly6C^+^ and Ly6C^−^ CD8^+^ T cells during ALI process with the treatment with MSCs.**

**Appendix Figure S2. t-SNE map of CD8^+^ T cells by mass cytometry.**

**a.** Dynamic changes of 13 clusters in different groups. P, PBS/PBS group; L3, LPS/PBS group at day 3; L7, LPS/PBS group at day 7; M3, LPS/MSC group at day 3; M7, LPS/MSC group at day 7. **b.** t-SNE map of markers in CD8^+^ T cells. Colors represent relative expression levels.

**Appendix Figure S3.** **Analysis of Cd8a^+^ T cells by scRNA-seq.**

**a.** Feature plot of Cd8a in lung T cells according to its normalized expression. **b.** Feature plot of Cd8a in selected lung Cd8a^+^ T cells according to its normalized expression. **c.** PPI network of DEGs in Ly6c^−^ Cd8a^+^ T cells between the LPS/PBS and LPS/MSC groups. **d.** Top 30 GO terms of DEGs downregulated in Ly6c^−^ Cd8a^+^ T cells between the LPS/PBS and LPS/MSC groups. **e.** Top 30 GO terms of DEGs upregulated in Ly6c^−^ Cd8a^+^ T cells between the LPS/PBS and LPS/MSC groups.
