# Supplementary material for: Mesenchymal stem cells alleviate LPS-induced acute lung injury by inhibiting the proinflammatory function of Ly6C+ CD8+ T cells
Source: Cell Death Dis. 2020 Oct 6;11(10):829. doi: 10.1038/s41419-020-03036-1 (PMC7538431; doi:10.1038/s41419-020-03036-1)
Supplement: Supplementary file 3 — Supplementary Table Legends [file 41419_2020_3036_MOESM3_ESM.docx]

**Appendix table legends**

**Appendix Table S1: List of 23 metal isotope-tagged antibodies for mass cytometry**

**Appendix Table S2: DEGs between Ly6c^-^ Cd8a^+^ T cells and Ly6c^+^ Cd8a^+^ T cells**

**Appendix Table S3: DEGs of Ly6c^+^ Cd8a^+^ T cells between the LPS/PBS group and the LPS/MSC group at day 7**

**Appendix Table S4: DEGs of Ly6c^-^ Cd8a^+^ T cells between the LPS/PBS group and the LPS/MSC group at day 7**

**Appendix Table S5: Top 10 of DEGs between Ly6c^-^ Cd8a^+^ T cells and Ly6c^+^ Cd8a^+^ T cells**

**Appendix Table S6: Top 10 of DEGs in Ly6c^+^ Cd8a^+^ T cells between the LPS/PBS group and the LPS/MSC group at day 7**

**Appendix Table S7: Top 10 of DEGs in Ly6c^-^ Cd8a^+^ T cells between the LPS/PBS group and the LPS/MSC group at day 7**
