# Supplementary figures and images for: Mesenchymal stem cells alleviate LPS-induced acute lung injury by inhibiting the proinflammatory function of Ly6C+ CD8+ T cells
Source: Cell Death Dis. 2020 Oct 6;11(10):829. doi: 10.1038/s41419-020-03036-1 (PMC7538431; doi:10.1038/s41419-020-03036-1)

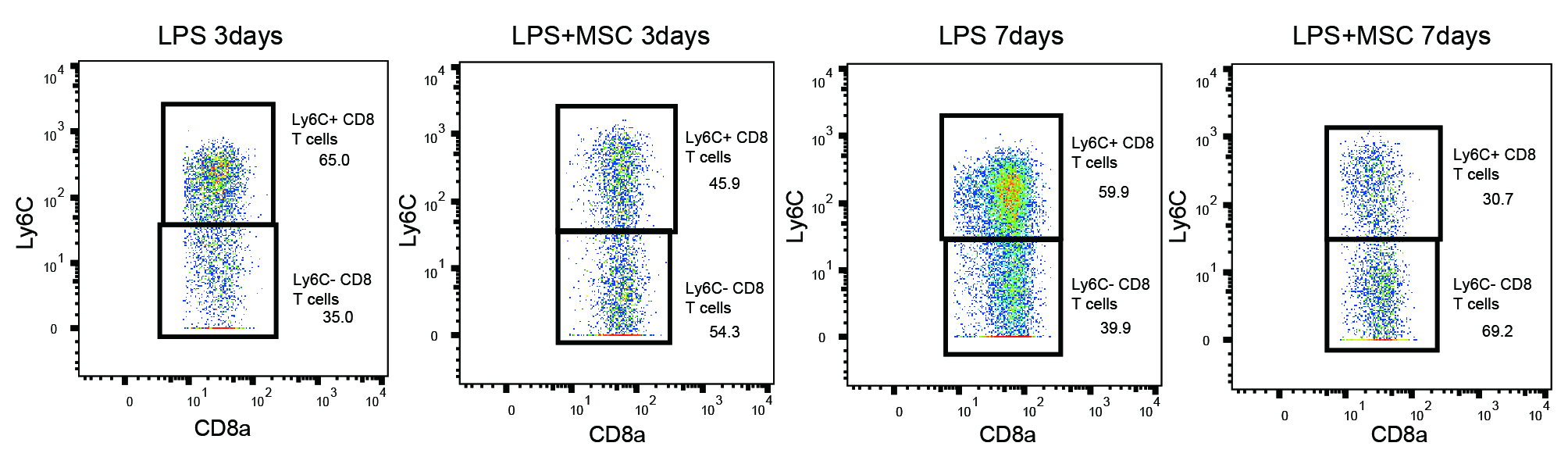

Supplement: Supplementary file 4 — supplementary Figure S1 [file 41419_2020_3036_MOESM4_ESM.tif]

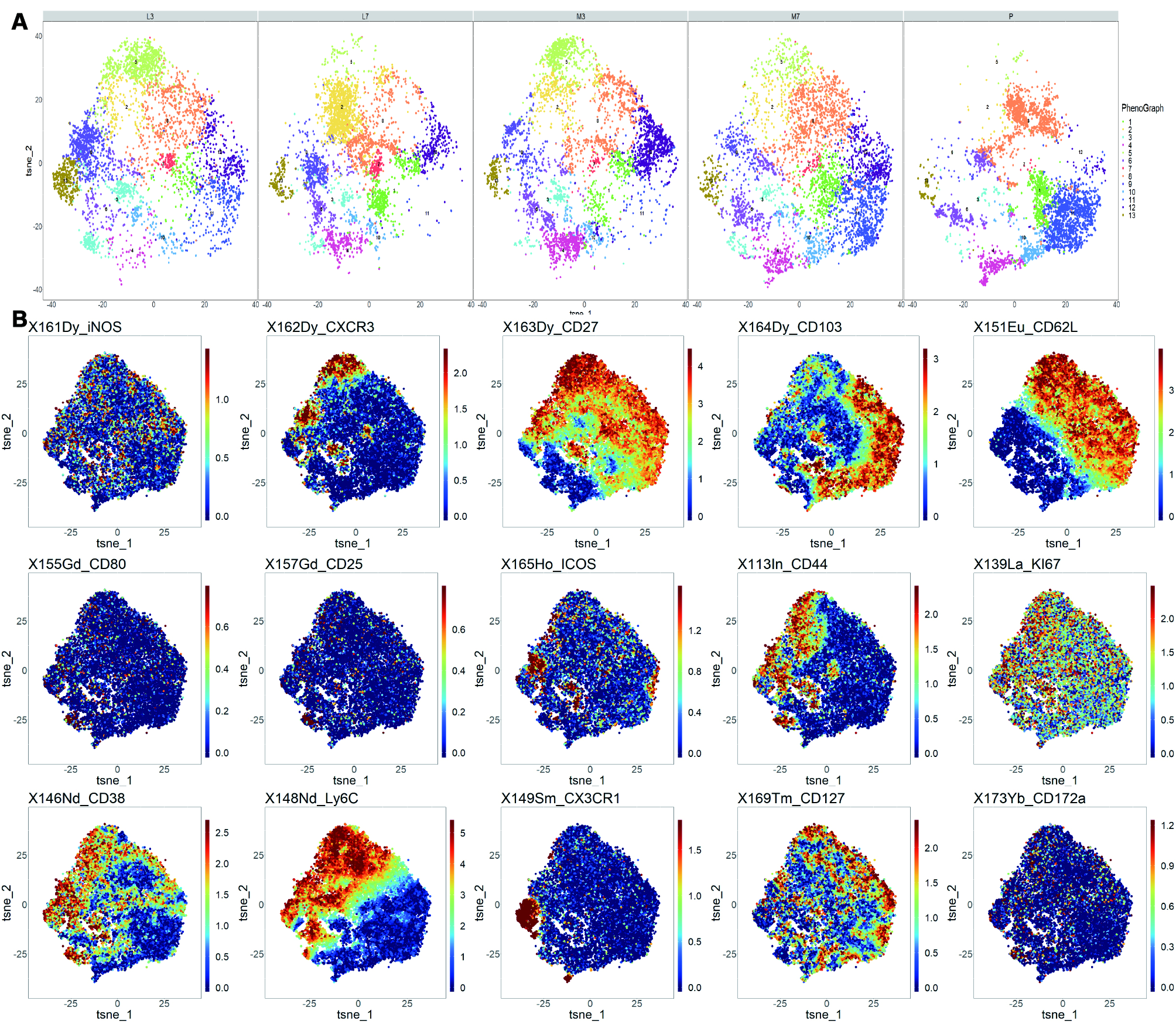

Supplement: Supplementary file 5 — supplementary Figure S2 [file 41419_2020_3036_MOESM5_ESM.tif]

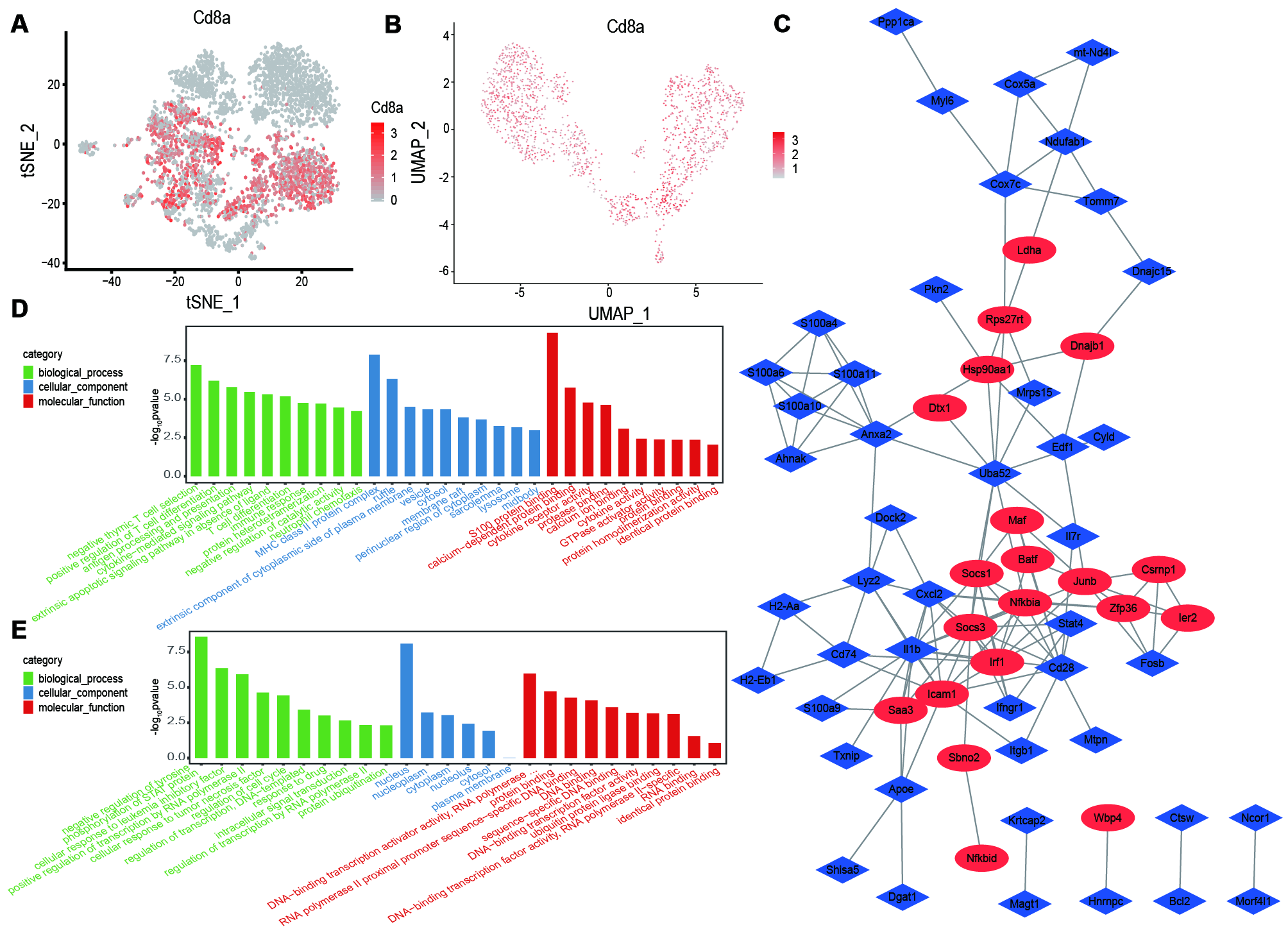

Supplement: Supplementary file 6 — supplementary Figure S3 [file 41419_2020_3036_MOESM6_ESM.tif]
